# Postoperative Rehabilitation Protocol After Isolated Meniscal Repair: A Systematic Review

**DOI:** 10.1177/23259671251357513

**Published:** 2025-07-23

**Authors:** Marc Daniel Bouchard, Matthew Macciacchera, Justin Gilbert, Darius Luke Lameire, Jihad Abouali

**Affiliations:** * Division of Orthopaedic Surgery, McMaster University, Hamilton, Ontario, Canada; † Division of Orthopaedic Surgery, University of Ottawa, Ottawa, Ontario, Canada; ‡ McMaster University, School of Medicine, Hamilton, Ontario, Canada; § Division of Orthopaedic Surgery, University of Toronto, Toronto, Ontario, Canada; ‖ Division of Orthopaedic Surgery, Michael Garron Hospital, Toronto, Ontario, Canada; ¶ University of Toronto Orthopaedic Sports Medicine, Toronto, Ontario, Canada; Investigation performed at University of Toronto, Canada

**Keywords:** meniscal repair, patient-reported outcome measures, postoperative outcomes, rehabilitation, weightbearing

## Abstract

**Background:**

The meniscus plays a critical role in knee stability and load distribution, with meniscal tears often resulting from trauma or degeneration. Rehabilitation protocols after meniscal repair lack standardization, particularly regarding weightbearing restrictions and their effect on outcomes.

**Hypothesis/Purpose:**

This systematic review hypothesizes that accelerated weightbearing protocols after isolated meniscal repair would lead to improved patient-reported outcomes and comparable failure rates relative to restricted rehabilitation protocols. The purpose was to evaluate the influence of different postoperative rehabilitation strategies on failure rates and functional outcomes after isolated meniscal repair.

**Study Design:**

Systematic review; Level of evidence, 4.

**Methods:**

Comprehensive searches of Embase, OVID Medline, and Emcare databases were conducted through November 2024. Studies were included if they reported on adult patients undergoing arthroscopic repair for isolated meniscal tears, described postoperative rehabilitation protocols, and had ≥10 months of follow-up. The primary outcome was repair failure (retears, revision surgery, or persistent symptoms). Secondary outcomes included patient-reported outcome measures (PROMs) and postoperative complications. Descriptive statistics summarized findings, with discrepancies resolved by a third investigator.

**Results:**

Ten studies (n = 313 patients) met the inclusion criteria. Most tears were medial (62%-93%) and primarily vertical or longitudinal, located in vascular zones. Rehabilitation protocols included accelerated/immediate weightbearing as tolerated (WBAT) (n = 7 studies), restricted weightbearing (n = 5 studies), and modified progressive programs (n = 1 study). Failure rates were 24.1% (accelerated WBAT), 28.3% (restricted), and 4.3% (modified progressive). PROMs (Lysholm and Tegner scores) were generally higher in accelerated WBAT protocols, with scores exceeding 85 and 7.2, respectively. Modified progressive rehabilitation yielded the lowest failure rate (4.3%) and favorable PROMs in the 1 study that utilized this method.

**Conclusion:**

The relationship between meniscal tear characteristics, repair techniques, and postoperative rehabilitation protocols plays a pivotal role in determining outcomes after isolated meniscal repair. Accelerated rehabilitation protocols may offer benefits such as faster recovery and improved patient satisfaction; nonetheless, they must be balanced against the increased risk of repair failure, particularly in complex tear patterns. Individualized rehabilitation protocols, accounting for tear characteristics, patient health, and surgical techniques, may optimize outcomes.

The medial and lateral menisci are a pair of C-shaped fibrocartilaginous structures found in the knee joint, sitting between the tibial and femoral articular surfaces.^24^ They play a critical role in knee stability and force distribution, acting to protect the articular surface and helping optimize knee function and convert load forces from axial to circumferential.^7,24^

Meniscal tears are extremely common and debilitating knee injuries, with an incidence of 70 per 100,000 people yearly.^8^ Patients often have pain, swelling, and stiffness, especially during knee flexion and extension.^12^ The size and severity of meniscal tears vary based on the mechanism of injury.^21^ Medial meniscal tears are considered generally more common than lateral tears, likely due to increased constraint from attachments to adjacent ligamentous structures.^22^ However, associated knee injuries can predispose the lateral meniscus to injury, such as an acute rupture of the anterior cruciate ligament (ACL).^8,22,25^ Conversely, medial meniscal tears are more common in chronic ACL-deficient knees.^22^ This is especially true when ACL reconstruction is delayed over a year from injury, as it serves to stabilize the knee by preventing anterior tibial translation.^22,25^ These patterns highlight the relationship between meniscal stability and knee biomechanics.^8,22,25,32^

Traumatic meniscal tears often occur when a rotational force is applied through the knee while the foot is firmly planted on the ground.^22,36^ Axial loading combined with rotation creates a shearing force through the joint, which can result in a tear to the meniscal fibrocartilage.^23,36^ Medial meniscal injuries are typically more severe because of the firm attachment to the tibial plateau.^23^ Current treatment guidelines favor surgical repair over partial meniscectomy, although reported failure rates^5^ are as high as 36.4%. The success of arthroscopic meniscal repair is highly variable, depending highly on tear location and the associated vascular implications.^23^ The meniscus is anatomically divided into vascular zones, first described by Cooper et al^11^ in 1990, transitioning from the well-vascularized red-red zone (outermost) to the poorly vascularized white-white zone (innermost).^9,11^ This explains the modern understanding that tears in the red-red zone exhibit better healing potential.^22,35^

In addition to vascularity, tear characteristics and the repair technique utilized (eg, inside-out, outside-in, or all-inside arthroscopic methods) may influence healing. Common meniscal tear patterns include vertical/longitudinal, radial, horizontal, bucket-handle, and complex/degenerative.^4^ Depending on the tear characteristics assessed on magnetic resonance imaging and/or intraoperatively, orthopaedic surgeons may tailor the repair method used.^29^ Meniscal repair is performed to preserve knee function and prevent long-term degenerative changes.^7,23^ Similar to other orthopaedic procedures, postoperative rehabilitation plays a pivotal role in determining both clinical and patient-reported outcomes.^9^

Despite advancements in surgical techniques, the variability in postoperative rehabilitation protocols presents a significant challenge. While many existing protocols recommend restricted weightbearing and limited range of motion (ROM),^29^ evidence supporting these restrictions is limited. Consequently, there is a need to evaluate whether an accelerated weightbearing protocol, compared with restricted weightbearing, is both safe and effective in promoting recovery and improving clinical outcomes. This systematic review aimed to assess current evidence on restricted versus accelerated postoperative rehabilitation after isolated meniscal repair. Tear characteristics and surgical techniques are also considered to better understand their association with outcomes. We hypothesized that accelerated weightbearing protocols after isolated meniscal repair would lead to improved patient-reported outcomes and comparable failure rates compared with restricted rehabilitation protocols.

## Methods

We conducted a systematic review in accordance with the PRISMA (Preferred Reporting Items for Systematic Reviews and Meta-Analyses) guidelines.^19^ In this review, “accelerated rehabilitation” refers to protocols that allow for full weightbearing early postoperatively, with progressive ROM exercises and minimal immobilization. “Restricted rehabilitation” includes protocols that limit weightbearing during the early postoperative period, may involve immobilization with a brace, and typically incorporate more gradual progression in ROM. Ethical approval was not required as this study was a systematic review of published literature.

### Search Strategy

A comprehensive literature search was performed using the electronic databases Embase, OVID Medline, and Emcare from database inception to the 47th week of 2024. Manual searches were performed to ensure the inclusion of all relevant literature by reviewing reference lists of pertinent articles. Medical Subject Headings terms (“meniscus” OR “menisci”) AND (“tear” OR “injury” OR “torn” AND (“repair” OR “operation” OR “surgery” OR “arthroscopy”) AND (“weightbearing” OR “weightbear”) AND (“rehabilitation” OR “rehab” OR “accelerated rehab” OR “restricted rehab” OR “progressive rehab” AND (“outcome” OR “failure rate” OR “re-tear rate”) were used as keywords. Boolean operators (eg, AND, OR) were used to refine the search strategy. Two reviewers (M.D.B. and M.M.) independently screened titles, abstracts, and full-text articles resulting from the searches. Any disagreements were addressed by discussion between the 2 reviewers and the senior author (J.A.) when necessary. In addition, all references cited in the identified reviews were manually searched for other articles not identified by the initial search strategy.

### Eligibility Criteria

The inclusion criteria for this review were as follows: (1) patients with isolated meniscal tears who underwent arthroscopic operative repair; (2) postoperative description of the rehabilitation protocol; (3) a minimum follow-up period of 10 months; (4) clinical studies; (5) adult patients aged ≥18 years; and (6) cohorts of at least 10 participants. There were no restrictions on the year of publication. All non-English studies, conference abstracts, case reports/series, and anecdotal or nonoriginal research, such as reviews, editorials, or commentaries, were excluded. In addition, studies were excluded if they presented data overlapping with another study using the same population, in which case the study with the longest follow-up period was considered.

### Data Extraction

The primary outcome of this study was the failure rate of meniscal repair, defined as the incidence of retears, revision surgery, or persistent symptoms necessitating further intervention. Secondary outcomes included patient-reported outcome measures (PROMs), with emphasis on changes exceeding the Minimal Clinically Important Difference (MCID), as well as postoperative complications.

Two reviewers (M.D.B. and M.M.) extracted data from eligible studies using a standardized data collection form on Microsoft Excel Version 16.90. Information collected included study characteristics (author, year of publication, level of evidence, study design, sample size, and length of follow-up), patient characteristics (age and sex), clinical variables (rehabilitation protocol, tear characteristics and side, and repair technique), and outcomes recorded (patient-reported retear rate).

### Quality Assessment

The Methodological Index for Non-randomized Studies (MINORS) score was used to assess the quality of studies included in the review, with a global ideal score of 16 for noncomparative studies and 24 for comparative studies. For this review, and consistent with other literature, a score of ≤8 was considered to be of poor quality, 9 to 14 moderate quality, and 15 to 16 good quality for noncomparative studies. These scores were ≤14, 15 to 22, and 23 to 24 for comparative studies, respectively.^28^

### Statistical Analysis

Given the heterogeneity in study designs and outcome measures, data were synthesized descriptively and presented as ranges when appropriate. All measures of variance were done using Microsoft Excel Version 16.90. Agreement between reviewers was evaluated using the Cohen kappa statistic (κ) at all screening stages. Agreement was classified a priori as follows: κ of 0.81 to 0.99 was considered nearly perfect agreement; κ of 0.61 to 0.80 was substantial agreement; κ of 0.41 to 0.60 was moderate agreement; 0.21 to 0.40 was fair agreement, and a κ value of ≤0.20 was considered slight agreement.^17^ Any disagreements were resolved through discussion with a third investigator and senior author (J.A.).

### Dealing With Missing Data

In accordance with the guidelines provided in the Cochrane Handbook for Systematic Reviews of Interventions, we contacted the authors of studies with missing data to obtain the desired information.^14^

## Results

### Identification of Eligible Studies

**Figure 1.**
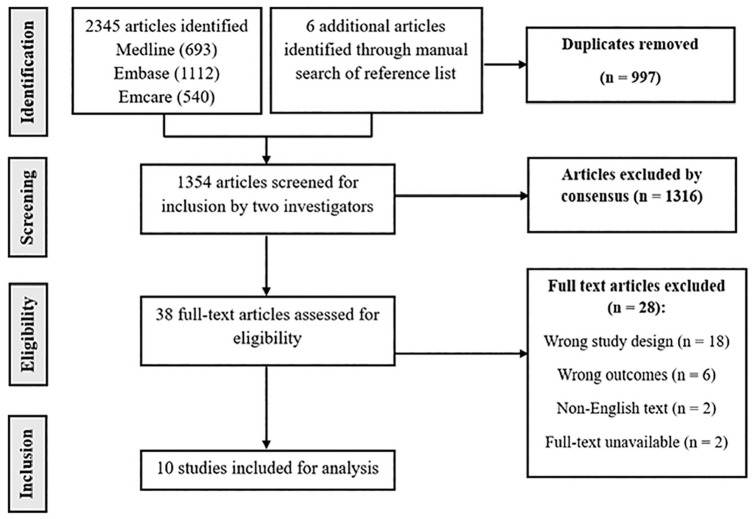
PRISMA Study Flow Diagram. PRISMA, Preferred Reporting Items for Systematic Reviews and Meta-Analyses.

A total of 2345 articles were initially identified through searches of the Embase, OVID Medline, and Emcare databases (Appendix Table A1), along with 6 additional citations from manual reference list reviews. After removing 997 duplicates, 1354 studies underwent title and abstract screening by 2 investigators (M.D.B. and M.M.). From this, 38 studies were selected for full-text review. The agreement between the reviewers was moderate at the title and abstract stage (κ = 0.58) and almost perfect at the full-text stage (κ = 0.84). After a thorough evaluation against the inclusion and exclusion criteria, 28 studies were excluded—18 studies were excluded for incorrect study design, 6 for reporting unrelated outcomes, 2 because the full-texts were unavailable, and 2 because they were not in English. Ten studies met the eligibility criteria for inclusion in this review (Figure 1).

In this review, 8 noncomparative studies^1,2,3,15,16,26,30,31^ and 2 comparative studies^6,20^ were included. Scores on the MINORS for noncomparative studies ranged from 13 to 16, with the majority being graded as having a good quality (15-16) according to our criteria (Appendix Table A2).^17^ This was mostly owing to a lack of unbiased study endpoints. No studies were graded as having a poor quality, and only 2 studies were graded as having a moderate quality by these same standards. Both comparative studies were of good quality, with scores of 23 out of 24 and 24 out of 24.

### Study and Patient Characteristics

A total of 741 knee surgeries were reported across the 10 included studies, of which 313 involved isolated meniscal repairs (Table 1). In studies that included patients undergoing concomitant ACL repairs, results were stratified to focus exclusively on the isolated meniscal repair cohort to ensure specificity in rehabilitation and outcome analysis. Most repairs were performed on medial menisci (Table 2), and the overall sex distribution was not consistently reported. Among the studies reporting sex distribution, the total female percentage was 32.7%. The mean patient age was 26.7 years (range, 14.6-43.7 years), and the mean follow-up duration was 42.5 months (range, 17-65.9 months).

**Table 1:** Patient and Study Characteristics*^*a*^*

Author (Year)	Study Design & Level of Evidence	MINORS Score	Total No. of Surgeries*^*b*^* (Isolated Meniscus)	Mean Age, Years (Range)	Mean Follow-up (Range), Months	Female, %
Barber^1^ (1994)	PC, 3	15	95 (34)	24.2 (14-45)	30.6 (12-89)	21
Barber & Click^2^ (1997)	PC, 3	15	65 (6)	26 (13-44)	38 (24-72)	22.2
Barber et al^3^ (2008)	PC, 4	16	41 (12)	28 (12-52)	30.7 (12-58)	26.8
Bryant et al^6^ (2007)	RCT, 1	24	100 (35)	25.4 (17-35)	28 (9-46)	38
Kocabey et al^15^ (2004)	RCS, 4	13	55 (23)	26.7 (13-50)	10.3 (4-24)	32.7
Kurzweil et al^16^ (2005)	PCS, 4	15	60 (12)	27 (13-53)	54 (36-70)	NR
Lind et al^20^ (2013)	RCT, 1	23	60 (60)	27.6 (18-50)	24 (NR)	31.7
Perkins et al^26^ (2018)	RC, 3	14	157 (62)	25.3 (12-36)	60 (NR)	43.3
Stein et al^30^ (2010)	RC, 3	16	81 (42)	33.3 (19-47)	77.6 (24-86)	34.6
Suganuma et al^31^ (2010)	Case control, 3	16	27 (27)	25.6 (18-45)	41.3 (25.2-70.8)	22.2
Total values			741 (313)	26.7 (14.6-43.7)	42.5 (17-65.9)	32.7

a MINORS, Methodological Index for Non-randomized Studies; NR, not reported; PC, prospective cohort; RC, retrospective cohort; RCS, retrospective case series; RCT, randomized controlled trial; PCS, prospective case series.

b Values in brackets represent the isolated meniscal repair population, in studies where concomitant ACL repairs were considered.

**Table 2:** Tear Characteristics, Operative Techniques, and PROS*^*a*^*

Author (Year)	Tear Type	Repair Technique	Weightbearing Restrictions	Postoperative Tegner	Postoperative Lysholm	Failure Rate in Isolated Repairs
Barber^1^ (1994)	93 medial	Inside-out repair	Group 1: NWB Group 2: WBAT, ROMAT	NR	NR	Group 1: 28.6 Group 2: 33.3
Barber & Click^2^ (1997)	89 medial, 11 lateral	Inside-out repair	WBAT, ROMAT	NR	NR	33.3
Barber et al^3^ (2008)	63 medial	All-inside repair (T-Fix)	WBAT, ROMAT	7.2 ± 2.9	87.4 ± 29.9	16.7
Bryant et al^6^ (2007)	Group 1: 78 medial, 70 posterior Group 2: 90 medial, 55 posterior	All-inside repair or Inside-out repair	WBAT in extension	NR	NR	22 (global failure rate)*^*b*^*
Kocabey et al^15^ (2004)	78 medial, 80 1-2 cm in length	All-inside repair (T-Fix)	WBAT in simple tear patterns	NR	NR	4.3
Kurzweil et al^16^ (2005)	62 medial, vertical/ longitudinal	All-inside repair (arrows)	Immediate WBAT*^*c*^*	NR	NR	42
Lind et al^20^ (2013)	70 medial 30 lateral	All-inside repair (arrows + T-Fix)	Group 1: Immediate WBAT Group 2: Restricted WB	Group 1: 4.5 ± 1.9 Group 2: 4.1 ± 2.1	NR	Group 1: 28.1 Group 2: 35.7
Perkins et al^26^ (2018)	Vertical/ longitudinal, peripheral	All-inside or inside-out repair	WBAT in extension (6/8 surgeons)	NR	NR	38.9 (global failure rate)*^*b*^*
Stein et al^30^ (2010)	Medial	Inside-out repair (sutures)	WBAT in extension, 4 weeks	5.46 ± 1.5	91.54 ± 8.95	14.3
Suganuma et al^31^ (2010)	Vertical	Inside-out repair	Group 1: WBAT week 8 Group 2: WBAT week 2	Group 1: 4.2 Group 2: 4.7	Group 1: 86.5 ± 7.8 Group 2: 97.1 ± 3.5	25.9

a Data are presented as mean ± SD or % unless otherwise indicated. NR, not reported; NWB, nonweightbearing; PROS, patient-reported outcomes; ROMAT, range of motion as tolerated; WBAT, weightbearing as tolerated.

b No mention of isolated meniscal repair retear rates.

c Rehabilitation slowed from accelerated to restricted after 10 patients because of early failures.

Study designs varied, and 8 of the 10 studies were nonrandomized, which included 5 cohort studies (2 retrospective^26,30^ and 3 prospective^1-3^), 2 case series (1 retrospective^15^ and 1 prospective^16^), and 1 therapeutic case-control study (Suganuma et al^3^). The remaining 2 studies by Bryant et al^6^ and Lind et al^20^ were randomized controlled trials (RCTs).

In studies where concomitant ACL repairs were included, the results were stratified to focus on the isolated meniscal repair cohort. In cases where this distinction was not made or where there was not a failure rate exclusive to isolated meniscal repairs, these data were not included in our pooled failure rate analysis but were referenced in discussing general rehabilitation trends. This ensured the findings were specific to the rehabilitation and outcomes of isolated meniscal repair procedures. Variability in reporting was noted for certain demographic and clinical variables.

### Tear Characteristics and Operative Techniques

Most meniscal tears involved the medial meniscus, with percentages ranging from^16^ 62% to^1^ 93% across studies. Tear types were predominantly vertical or longitudinal, often located in the posterior horn or peripheral regions in the red-red or red-white zones. The meniscal repair technique was described in all 10 studies. An all-inside technique was utilized in 4 studies, of which 2 studies^3,15^ used FasT-Fix devices, 1 study used meniscal Bionx Arrows,^16^ and 1 study used both (Lind et al^20^). An inside-out technique was used in 4 studies with a combination of different suture types.^1-3,31^ Two studies^6,26^ used a combination of all-inside and inside-out repairs. Among studies reporting repair techniques, inside-out repairs were used more frequently for medial and larger tears, whereas all-inside repairs were often employed for shorter and simpler tear patterns (Table 2).

### Patient-Reported Outcome Measures

Postoperative Tegner and Lysholm scores were inconsistently reported across studies. Of the included studies, 4 reported^3,20,30,31^ postoperative activity levels using the Tegner Activity Scale, with levels ranging^20^ from 4.1 ± 2.1 to^3^ 7.2 ± 2.9. Three studies^3,30,31^ utilized the Lysholm Knee Scoring Scale to assess postoperative outcomes, all with a score >85 postoperatively. Suganuma et al^31^ reported the highest Lysholm scores (97.1 ± 3.5) in the group with delayed weightbearing as tolerated (WBAT) rehabilitation protocol (Table 2).

PROMs were generally more favorable after all-inside repairs. Barber et al^3^ reported a mean postoperative Tegner score of 7.2 ± 2.9 after all-inside repairs with WBAT protocols. Conversely, Tegner scores were lower in patients undergoing inside-out repairs, with Stein et al^30^ reporting a mean score of 5.46 ± 1.5 and Suganuma et al^31^ reporting a score of 4.2 despite an otherwise unrestricted WBAT protocol (Table 2).

### Weightbearing Protocols and Outcomes

Weightbearing protocols after meniscal repair varied widely across the 10 studies included, influenced by both the repair technique and the meniscal tear characteristics. Immediate WBAT was permitted in 7 studies, most of which included all-inside repair techniques.^1-3,16,20,26,31^ However, studies involving inside-out repairs more commonly included restricted weightbearing or nonweightbearing (NWB) protocols, which were employed in 5 studies.^1,20,26,30,31^ Lind et al^20^ directly compared immediate WBAT with restricted weightbearing protocols and found no significant difference in failure rates (28.1% vs 35.7%, respectively, *P* > .05). However, the effect of weightbearing restrictions on failure rates was not consistent across studies. Barber^1^ found lower failure rates in the WBAT group (33.3%) compared with the NWB group (28.6%), when ROM was not restricted (Table 2).

A notable exception was the study by Kocabey et al,^15^ where a modifiable, progressive rehabilitation program tailored to tear length yielded excellent clinical results (Tables 2 and 3). In this study, anteroposterior longitudinal meniscal tears <3 cm in length were restricted in knee flexion from 0° to 90° for 3 weeks, progressing to 0° to 125° flexion from weeks 3 to 6. If these tears were >3 cm, the knee was immobilized at 0° flexion using a locked, long-leg hinged knee brace for full weightbearing during the first 3 weeks. In both groups, all restrictions on knee flexion (0°-125° active ROM) were terminated between weeks 6 to 8. Also, 22 of 23 patients who had meniscal repair achieved good clinical outcomes, with only 1 patient (4.3%) requiring revision surgery.

**Table 3:** Overall Failure Rates of Different Rehabilitation Protocols After Isolated Meniscal Repairs*^*a*^*

Rehabilitation Protocol	Studies Reporting	No. of Isolated Meniscal Tears	Total No. of Failures	Overall Isolated Meniscal Repair Failure Rate
Accelerated	Barber,^1^ Barber & Click,^2^ Barber et al,^3 *b*^Kurzweil et al,^16^ Lind et al,^20^ Suganuma et al^31^	87	21	24.1
Restricted	Barber,^1^ Lind et al,^20^ Stein et al,^30^ Suganuma et al^31^	106	30	28.3
Modified, progressive	Kocabey et al^15^	23	1	4.3

a Data are presented as % unless otherwise indicated.

b Rehabilitation slowed from accelerated to restricted after 10 patients due to early failures.

Protocols allowing for early WBAT were associated with improved PROMs. Suganuma et al^31^ reported significantly higher postoperative Tegner scores (4.7 vs 4.2) and Lysholm scores (97.1 ± 3.5 vs 86.5 ± 7.8) in patients who began WBAT at week 2 compared with those who initiated WBAT at week 8 (*P* < .05). Lind et al^20^ also reported higher postoperative Tegner scores (4.5 vs 4.1) in the immediate WBAT group at the final follow-up; however, this difference was not significant (*P* > .05).

### Failure Rates Across Rehabilitation Protocols and Techniques

Failure rates showed substantial variability across studies and rehabilitation protocols, ranging from 4.3% to 42% in isolated meniscal repairs (Table 2). Immediate WBAT protocols yielded mixed results. While Lind et al^20^ observed lower failure rates in the WBAT group (28.1%) compared with restricted WB (35.7%), Kurzweil et al^16^ reported a significantly higher failure rate (42%) during the early phase of an accelerated rehabilitation protocol, which was subsequently modified to include restricted WBAT.

In contrast, restricted weightbearing protocols were not consistently protective. Perkins et al^26^ and Barber^1^ noted failure rates of 38.9% and 28.6%, respectively, despite limiting weightbearing and ROM early in the rehabilitation process. It is important to note that the failure rate of 38.9% reported by Perkins et al^26^ is a global failure rate of all meniscal repairs in the study. This includes menisci repaired with concomitant ACL reconstruction, as there was no specific failure rate reported for isolated repairs only.

The failure rates for different rehabilitation protocols after isolated meniscal repairs are summarized in Table 3. Across the studies, the overall failure rate varied significantly depending on the rehabilitation approach. Accelerated rehabilitation protocols were reported in 7 studies, encompassing 87 isolated meniscal repairs. Note that 1 study (Perkins et al^26^) reported a global failure rate and not a failure rate for isolated tears and was therefore not included in this table or calculation. These studies demonstrated 21 failures, resulting in an overall failure rate of 24.1%. Notably, 1 study (Kurzweil et al^16^) transitioned from accelerated to restricted rehabilitation after observing early failures in the first 10 patients. Restricted rehabilitation protocols were reported in 5 studies, with 106 isolated meniscal repairs and 30 documented failures, yielding a higher overall failure rate of 28.3%. Note that the study by Bryant et al^6^ reported only global failure rates and was not included in the calculation. This approach was generally more protective, particularly for complex tear types, but failure rates remained high. A modified, progressive rehabilitation protocol was described in 1 study (Kocabey et al^15^), which included 23 isolated meniscal repairs. This approach showed the lowest failure rate, with only 1 failure recorded (4.3%) (Table 3).

## Discussion

This systematic review evaluated outcomes after isolated meniscal repair and the effect of varying rehabilitation methods, with a specific focus on postoperative weightbearing protocols, tear characteristics, and repair techniques. Both accelerated and restricted rehabilitation protocols after isolated meniscal tear repair led to comparable failure rates. However, the small number of studies and limited sample sizes within each rehabilitation group restrict the generalizability of these findings. The heterogeneity in tear types, repair techniques, and study designs also limits direct comparisons between protocols, and these factors likely contributed to the variability in reported failure rates across studies. A patient-tailored rehabilitation protocol that takes into account factors such as weightbearing restrictions, tear length (> or <3 cm), and repair technique may be most beneficial, as demonstrated by Kocabey et al.^15^ The results of this study highlight a lack of consensus on the optimal approach to postoperative rehabilitation, underscoring the need for individualized protocols tailored to specific tear patterns and repair methods.

### Comparison With Existing Literature

The findings of this review align with previous systematic reviews, such as those by VanderHave et al^34^ and You et al.^38^ These studies also highlight the lack of consensus in rehabilitation protocols after meniscal repair and found that direct comparisons cannot be made without controlling for confounding variables such as size, location, and type of tear. However, this review differs by narrowing the scope to include isolated meniscal repairs only, providing a more focused analysis of the rehabilitation considerations for this cohort.

### Variability in Tear Pattern and Repair Technique

The variety of meniscal tear types and repair techniques underlines the possible difficulty in establishing a universal rehabilitation protocol. Meniscal healing is highly dependent on adequate vascular supply and biomechanical stability.^23^ Peripheral tears in the red-red or red-white zones benefit from their proximity to vascularized regions, which may support the use of accelerated WBAT protocols in these cases. This is evidenced by the low reported failure rate (16.7%) in Barber et al^3^ study. Conversely, tears in avascular zones or those extending into the central meniscus may require more cautious approaches, as they are more reliant on the mechanical stability provided by sutures.^9,11,22,35^ Evidence from Kurzweil et al^16^ highlights this potential for early failure in accelerated protocols, especially when dealing with complex tear types. They report the highest failure rate (42%) in this study, where the accelerated rehabilitation protocol was subsequently modified to include restricted WBAT after 5 of the first 10 patients experienced a failure of the repair. Similarly, Perkins et al^26^ underscores the high failure rates associated with bucket-handle tears, irrespective of repair strategy. They reported a 38.9% failure rate for patients treated with all-inside and inside-out techniques, suggesting that complex tear patterns may be more susceptible to failure, regardless of rehabilitation protocol. Nonetheless, this failure rate represented all meniscal repairs done in the study, not just isolated repairs, which suggests that the inclusion of concomitant procedures or more severe cases could have influenced the overall outcomes.

It is also important to note that restricted weightbearing protocols were not consistently protective against repair failure, and the variability in reported failure rates may reflect underlying selection bias. Given that most included studies were noncomparative, it is possible that surgeons preferentially assigned patients with more complex or higher-risk tears to restricted protocols while allowing smaller, less complex tears to follow accelerated rehabilitation. This was evidenced by studies like Lind et al,^20^ which reported a significant failure rate of 35.7%, even with restricted protocols. This suggests that factors beyond weightbearing status, such as tear complexity, repair technique, and patient adherence, may play a more critical role in determining outcomes.

### Patient-Reported Outcomes and the Importance of MCID

The inclusion of PROMs—such as the Tegner Activity Scale and the Lysholm Knee Score—provides valuable insights into functional recovery. However, their inconsistent use across studies makes direct comparisons difficult. The MCID helps quantify the clinical relevance of observed changes. The Lysholm Knee Score MCID is reported in the literature^9,27,37^ as 10.1 to 11.1. The MCID for the Tegner Activity Scale is reported^27^ as 0.9. Three of the 4 studies reporting PROMs^3,20,31^ in this review demonstrated pre- and postoperative improvements surpassing the MCID. This suggests that meniscal repairs, when combined with appropriate rehabilitation protocols, result in clinically meaningful improvements in patient outcomes. Studies implementing early WBAT consistently reported higher Lysholm scores (exceeding^3,31^ 85) and Tegner activity scores (up to^3^ 7.2), reflecting improved activity levels and knee function. Notably, Suganuma et al^31^ highlighted significantly better Lysholm scores in early WBAT groups compared with delayed weightbearing protocols (86.5 ± 7.8 vs 97.1 ± 3.5), reinforcing the functional benefits of early mobilization. However, these advantages must be weighed against the increased failure risks observed in certain populations, particularly those with complex or medial tears. The Tegner scores in this group did not surpass the MCID pre- to postoperatively, possibly reflecting limitations in activity level recovery despite functional improvements.

Interestingly, the restricted weightbearing group in the Lind et al^20^ study reported a decrease in Tegner scores from 5.1 ± 2.4 to 4.1 ± 2.1 postoperatively. Restricted weightbearing might hinder the patient’s ability to progressively load and mobilize the affected area, leading to a slower recovery of function and physical activity levels. This restriction could result in a decline in the ability to participate in higher-demand activities, reflected in the lower Tegner scores. Furthermore, the severity of tear patterns in this cohort may have been more substantial, which could influence postoperative recovery and activity levels, compounding the effect of restricted rehabilitation.^10^ For instance, it has been suggested that more severe tears, which completely disrupt the longitudinal loop integrity of the meniscus, permit more restricted rehabilitation due to concerns of stressing the repair site.^10^ Thus, more disruptive tears employing restricted rehabilitation more often may muddle the actual effect that restricted rehabilitation has on recovery and outcomes in isolated meniscus repair.

The lack of uniform pre- and postoperative PROM data limits the ability to draw definitive conclusions about the efficacy of accelerated rehabilitation protocols after meniscal repair. This is especially relevant when considering the known variation in tear pattern severity. The development and standardized use of PROMs in future high-quality RCTs will be crucial to refine these strategies and improve clinical care for patients undergoing isolated meniscal repair.

### Modifiable Rehabilitation Program

In the existing literature, longer tears (>2.5 cm) and tears with a meniscal rim width exceeding 3 mm have been associated with higher failure rates.^13,16,20^ This emphasizes the need for tailored postoperative rehabilitation strategies in such cases. Modifiable protocols may offer the flexibility to address risk factors for postoperative complications. The study by Kocabey et al^15^ underscores the potential for individualized rehabilitation protocols, particularly in long or high-risk tears. In this study, patients with tears >3 cm underwent knee immobilization at 0° flexion using a locked, long-leg hinged knee brace for full weightbearing during the first 3 weeks postoperatively. From weeks 3 to 6, motion was gradually increased, allowing for 0° to 125° flexion. For tears <3 cm, knee immobilization at 0° flexion was achieved using a locked, long-leg hinged knee brace for full weightbearing, while passive motion from 0° to 90° flexion was encouraged via a continuous passive motion (CPM) device during the first 3 weeks. Between weeks 3 and 6, active knee flexion up to 90° was permitted, alongside passive knee flexion up to 125° using the CPM device. Patients with complex or radial tears followed comparable rehabilitation protocols but experienced slightly delayed progressions in ROM and weightbearing. This individualized approach resulted in excellent outcomes, with 96% of patients achieving successful healing and only 4% requiring revision surgery. However, it is important to note that the heterogeneity in tear characteristics likely contributed to these outcomes, as shorter or less complex tears inherently carry a lower risk of failure. Nonetheless, these findings highlight the potential benefits of customizing rehabilitation protocols to optimize healing for various meniscal tear presentations.

Postoperative rehabilitation protocol must also be considered along with advancements in surgical instrumentation/techniques. As the treatment of more complex meniscal injury patterns improves, accelerated rehabilitation may become a consideration in this patient group as well. Early movement can offer several advantages, including the prevention of muscle atrophy and stiffness, optimizing the healing environment for the meniscus, and enhancing patient satisfaction by reducing recovery time.^10,22^ Similarly, accelerated protocols promote faster recovery, improved joint health, and reduced risks of complications like deep vein thrombosis or pulmonary embolism by promoting early mobilization.^18,33^ In fact, 1 patient in the Kurzweil et al^16^ study developed a pulmonary embolism postoperatively after the protocol was switched from accelerated to restricted. It is well known that the risk of developing a deep vein thrombosis is increased in surgical patients; thus, early mobilization should be recommended when clinically appropriate.^18,33^ However, this risk must be balanced with the possibility of increased failure, as also evidenced by Kurzweil et al,^16^ which had the highest reported failure rate at 42% and saw failures in 5 of the first 10 patients. This suggests that while early mobilization offers clear benefits, a careful balance must be struck between accelerating rehabilitation and ensuring that the patient’s healing recovery is not jeopardized.

### Limitations and Future Directions

Several limitations of this review must be acknowledged. First, there is a lack of recent clinical studies including patients who underwent isolated meniscal repair. Only 1 of the included studies in this review was conducted in the past decade. This gap in the literature calls into question the relevance of many of the included studies to current clinical practice. There is a need for up-to-date, high-quality clinical studies to allow for evidence-based rehabilitation protocols after meniscal repair. Second, the degree of data heterogeneity between the included studies precluded any weighted mean or pooled data synthesis. This was present in both clinical outcomes and PROMs. Finally, the predominance of non-randomized studies with moderate population sizes limits the generalizability of our findings. It is possible that surgeons preferentially assigned patients with more complex or higher-risk tears to restricted protocols, while allowing smaller, less complex tears to follow accelerated rehabilitation. These biases could have affected the outcomes and precluded any definitive conclusions on the noninferiority of accelerated protocols. RCTs with greater patient recruitment and standardized rehabilitation protocols are needed to provide more definitive evidence.

Future research should prioritize the development of standardized, evidence-based rehabilitation protocols that account for variations in tear pattern and repair technique, with consideration of patient-specific factors. RCTs comparing early versus delayed WBAT across different tear patterns may be particularly useful to clarify the efficacy of accelerated rehabilitation. In addition, studies should incorporate validated PROMs with established MCIDs to facilitate meaningful comparisons and ensure clinical relevance. Advancing our understanding of the biomechanical and biological factors influencing meniscal healing will also be critical. Finally, the integration of modifiable, patient-centered rehabilitation programs, as exemplified by Kocabey et al,^15^ warrants further exploration to validate their effectiveness in diverse patient populations.

## Conclusion

Meniscal tear characteristics, repair techniques, and postoperative rehabilitation protocols play a pivotal role in determining outcomes after isolated meniscal repair. This review demonstrated comparable failure rates in both accelerated and restricted rehabilitation protocols. Although the results suggest that accelerated rehabilitation may offer benefits such as faster recovery, reduced risk of complications (eg, deep vein thrombosis), and improved patient satisfaction, these findings are exploratory and cannot definitively confirm noninferiority. The increased risk of repair failure in patients with complex tear patterns underscores the importance of tailoring rehabilitation strategies based on tear characteristics, surgical techniques, and individual patient factors. Individualized protocols, accounting for the severity of the injury, the patient’s overall health, and surgical technique, may optimize patient outcomes and minimize the risk of failure. Future RCTs with standardized rehabilitation protocols and validated PROMS are essential to refine these strategies and establish evidence-based guidelines for optimal recovery.

## Appendix

**Table A1:** MeSH Terms and Boolean Operators Used to Develop Search Strategy from Embase, Emcare, and OVID Medline Databases

Database: Ovid MEDLINE(R) ALL <1946 to November 20, 2024>
Search Strategy:
**1**. exp Menisci, Tibial/ or exp Tibial Meniscus Injuries/ or exp Meniscus/ (10583)
**2**. (Meniscus or menisci).mp. (20239)
**3**. 1 or 2 (20239)
**4**. (repair* or surg* or operation* or meniscectomy or arthosco*).mp. (446618)
**5**. exp Weight-bearing/ or weightbear*.mp or "Weight bear*".mp or weight-bear*.mp or "retear rate*".mp. or "failure rate*".mp. or "accelerated rehab*".mp. or "restricted rehab*".mp. or "progressive rehab*".mp. or "Outcome and Process Assessment, Health Care"/ or *"Outcome Assessment, Health Care"/ (126104)
**6**. (tear* or injur* or torn).mp (1542646)
**7**. 3 and 4 and 5 and 6 (693)
Database: Embase <1974 to 2024 November 20>
Search Strategy:
**1**. exp knee meniscus/ or exp knee meniscus rupture/ (21578)
**2**. (Meniscus or menisci).mp. (28722)
**3**. 1 or 2 (28722)
**4**. (repair* or surg* or operation* or meniscectomy or arthosco*).mp. (6066500)
**5**. exp weight bearing/ or weightbear*.mp. or "Weight bear*".mp. or weight-bear*.mp or "retear rate*".mp. or "failure rate*".mp. or "accelerated rehab*".mp. or "restricted rehab*".mp. or "progressive rehab*".mp. (85642)
**6**. (tear* or injur* or torn).mp (2184878)
**7**. 3 and 4 and 5 and 6 (1112)
Database: Ovid Emcare <1995 to 2024 Week 47>
Search Strategy:
**1**. exp knee meniscus/ or exp knee meniscus rupture/ (10157)
**2**. (Meniscus or menisci).mp. (11850)
**3**. 1 or 2 (11850)
**4**. (repair* or surg* or operation* or meniscectomy or arthosco*).mp. (1265934)
**5**. exp weight bearing/ or weightbear*.mp. or "Weight bear*".mp. or weight-bear*.mp. or "retear rate*".mp. or "failure rate*".mp. or "accelerated rehab*".mp. or "restricted rehab*".mp. or "progressive rehab*".mp. (34638)
**6**. (tear* or injur* or torn).mp (596703)
**7**. 3 and 4 and 5 and 6 (540)

**Table A2:** MINORS Score Calculations Quality Assessment of All Included Papers in the Review*^*a*^*

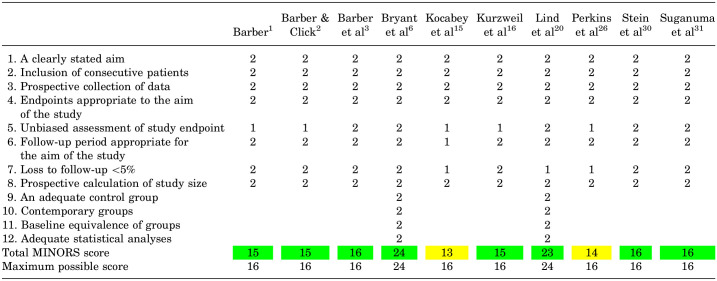

a MINORS, Methodological Index for Non-randomized Studies.

Legend (Total MINORS Score):  poor quality;  moderate quality;  good quality.
